# Current Developments in the Treatment of Postpartum Depression: Zuranolone

**DOI:** 10.5152/eurasianjmed.2024.24409

**Published:** 2024-10-01

**Authors:** Dogancan Sonmez, Cicek Hocaoglu

**Affiliations:** 1 Rize State Hospital, Clinic of Psychiatry, Rize, Türkiye; 2 Department of Psychiatry, Recep Tayyip Erdoğan University Faculty of Medicine, Rize, Türkiye

**Keywords:** Allopregnanolone, GABA, neuroactive steroids, postpartum depression, treatment, zuranolone

## Abstract

Pregnancy is a period in a woman’s life during which she experiences physiological, psychological, and social changes. These changes can lead to various mental illnesses, including postpartum depression (PPD), which is common during the perinatal period. Postpartum depression is a significant cause of morbidity and mortality for both the mother and baby. A peripartum-onset major depressive episode is defined as PPD when it occurs during pregnancy or up to 4 weeks postpartum. The frequency of this condition is extremely high. Its etiology is influenced by biological, psychological, and sociocultural factors. Depressed mood, anhedonia, feelings of guilt, irritability, lack of concentration, psychomotor agitation or retardation, sleep disturbance, and changes in appetite and weight can all be symptoms of PPD. There are various treatment options available, many of which are adapted from those used for major depression. Selective serotonin reuptake inhibitors, serotonin-noradrenaline reuptake inhibitors, tricyclic antidepressants, estradiol, progesterone, psychotherapies, electroconvulsive therapy, and brexanolone can be used to treat PPD. In addition, the newest drug approved by the FDA (Food and Drug Administration) for this condition is oral zuranolone. This review aims to analyze recent developments on zuranolone, the latest drug approved by the FDA for PPD, based on current studies.

**Main Points**

- PPD is one of the most important causes of mortality and morbidity during pregnancy and the subsequent period.
- Zuranolone received FDA approval for the treatment of PPD on August 4, 2023.
- Zuranolone is the first oral allopregnanolone agonist indicated for the treatment of PPD.
- The most important advantage of zuranolone for patients with PPD is that it offers a rapid and continuous treatment option.

## Introduction

Pregnancy is a crucial period in a woman’s life, during which she undergoes physiological, psychological, and social changes that require adaptation. These changes can cause various mental illnesses in women during pregnancy, delivery, and postpartum, including PPD.^1^ PPD is a common psychiatric disorder during the perinatal period and poses a significant risk to the mother and baby’s health and well-being.^2^ PPD is a common condition that can be overlooked in clinical practice. It has a distinct clinical presentation and etiology compared to other mood disorders.^3^ It can be added that the etiology and treatment of PPD have not been fully elucidated, making it different from other depressive disorders. It can be stated that pregnant and delivering women do not consult a physician or do not use the recommended treatment due to stigma, side effects of medications, and their desire to breastfeed. Fast and reliable treatment options are promising because of new approaches in the etiology of PPD in recent years. PPD is associated with high suicide rates^4^ and can have a negative impact on the quality of life of the mother, baby, and other family members.^3^ In this review, we will present the current literature on Zuranolone, the most recently approved treatment for PPD (Table 1).

### Postpartum Depression

Postpartum depression is a prevalent condition, but its definition varies. According to DSM-5-TR, PPD is a major depressive episode that occurs during pregnancy or up to 4 weeks after birth.^5^ International Classification of Disease (ICD-11), another common diagnosis and classification system, associates mental disorders that begin within 6 weeks after delivery with the puerperium.^6^ PPD is a significant complication during and after pregnancy, with a reported global prevalence rate of 14%.^7^ In the United States (US), it is the primary cause of pregnancy-related fatalities.^8^ According to recent data from the United Kingdom, completed suicide in the first year postpartum is the leading direct cause of maternal death.^9^

Postpartum depression is influenced by biological, psychological, and sociocultural factors.^10,11^ The etiology of PPD varies across cultures, with different psychosocial factors playing a role. While the postpartum period is significant in many cultures, traditional practices performed during this time can have both positive and negative effects on the mother and baby’s health. Some traditional practices may contribute to the development of PPD.^12^ PPD risk factors include a lack of social support, history of depression, young age, childcare problems, stressful life events, motherhood blues, negative marital relationships, poor marital relationships, changes in body image, low socioeconomic status, and undesired pregnancy.^13^ Physiological changes during pregnancy can increase the frequency of depression in pregnant women. Depression symptoms may increase in women during pregnancy and the postpartum period due to physical changes such as weight gain, nausea, and changes in respiratory capacity. These changes can lead to dissatisfaction and may contribute to the development of depression. This highlights the need for healthcare professionals to monitor and support women during this time.^14^ Hormonal changes occurring during and after delivery also play an important role in the etiology of PPD. Significant differences occur in the levels of steroid and peptide hormones in the body during pregnancy and the postpartum period. This is considered one of the most important hypotheses in the etiology of PPD. Fluctuations in these hormone levels include hormones such as estradiol, corticosterone, cortisol, progesterone, Corticotropin-Releasing Hormone (CRH), and oxytocin and occur in different pregnancy periods and with different profiles. The levels of these hormones increase significantly and tend to decrease with placental excretion.^15^ In a placebo-controlled study with biphasic estradiol, a positive correlation was found between decreased estradiol levels and increased Hamilton Depression Rating Scores, and it was reported that this was correlated with increased serotonin transporter levels in the neocortex in women. In this case, it probably causes a decrease in serotonin.^16^

In another study, prenatal androgen and estrogen levels were found to be associated with moderate PPD. These findings indicate that alterations in maternal and fetal steroid hormone production play a role in the development of PPD.^17^ By examining epigenetic modifications, changes in DNA methylation patterns have been detected in PPD.^18^ In neurobiological studies, neuroplastic changes occur in the prefrontal cortex and hippocampus in the postpartum period, regardless of mood. For example, total brain volume decreases and returns to normal levels in the 6th month after delivery. This may contribute to some women’s vulnerability to PPD.^19^

### Neurosteroid and GABAergic Signaling Hypothesis

Research on the pathophysiology of PPD has examined many hypotheses. These hypotheses provide several guiding theories for understanding the root causes of PPD. During pregnancy and the postpartum period, women’s hormone levels undergo major changes.^20^ Reproductive hormones, especially estrogen and progesterone, undergo great fluctuations during this process. These hormonal fluctuations can affect the nervous system and emotional regulation. Postpartum depression is thought to be related to hormonal changes. The Hypothalamus-Pituitary-Adrenal (HPA) axis is an important system that regulates stress responses. It is thought that this axis becomes dysregulated during pregnancy and the postpartum period and may lead to an excessive stress response. This may contribute to PPD symptoms.^21^ Gamma aminobutyric acid (GABA) is an important inhibitory neurotransmitter in the central nervous system. Changes in synaptic transmission of GABA can cause emotional regulation problems. During pregnancy and after delivery, the immune system undergoes various changes. These immune system changes can lead to increased inflammation and an increased risk of PPD. Inflammatory processes have been associated with PPD symptoms.^20-24^ Fluctuations in reproductive hormones during pregnancy and subsequent periods can cause changes in neurotransmitter levels, including serotonin, norepinephrine, dopamine, GABA, and glutamate. This situation also affects the HPA axis, which plays a crucial role in the body’s response to stress.^25^ The GABAergic signaling hypothesis is a hypothesis that has been associated with PPD and other emotional dysregulations. This hypothesis suggests that the GABA system, particularly allopregnanolone levels, plays an important role in the development of PPD. Allopregnanolone acts as a neuroactive metabolite of progesterone. Allopregnanolone is a potent modulator that affects GABA-A receptors in the brain. GABA-A receptors detect the inhibitory neurotransmitter GABA, which plays an important role in the communication of nerve cells. Therefore, allopregnanolone may have an effect on emotional regulation by regulating neural communication. The GABA system plays a role in regulating stress responses. Stress reactivity has been associated with GABA signaling disorders. That is, changes in the GABA system may affect stress responses and contribute to PPD symptoms. There is a hypothesis suggesting that some individuals may be more sensitive to GABA signaling, increasing the risk of PPD. This may be associated with genetic factors. Genetic variations may create a link between effects on the GABA system and the development of PPD.^26^ The study by Maguire and Mody^27^ represent important research examining the effects of genetic changes in GABA-A receptors using mouse models of PPD-like symptoms. This study was designed to understand the relationship of the GABA system and GABA-A receptors to the pathophysiology of PPD. According to the research results, female mice exhibited normal behavior throughout pregnancy. However, in the postnatal period, they showed signs of hyper-activation in the prefrontal cortex and amygdala, increased stress responses, and symptoms of depression. Additionally, they failed to exhibit maternal behaviors.^27^

The administration of allopregnanolone corrected the behavioral changes observed in the mice during the experiment. This outcome represents a significant advancement in the potential use of allopregnanolone for treating PPD.^28^

### Treatment Approaches

The treatment of PPD has been extensively studied in the literature. Numerous treatments have specifically been approved for PPD, taking inspiration from major depression treatment. Available treatment options include medications like selective serotonin reuptake inhibitors, serotonin-noradrenaline reuptake inhibitors, tricyclic antidepressants, estradiol, progesterone, psychotherapies, electroconvulsive therapy, brexanolone, and zuranolone. Only two drugs, brexanolone and zuranolone, have been approved by the FDA. Brexanolone was the first drug to receive approval in 2019, while zuranolone became the first oral drug to be approved in 2023.^29-31^

### Zuranolone

Recent studies on the pathophysiology of PPD have prompted research into synthetic neuroactive steroids and their analogues. These compounds can modulate GABA-A receptors and show promise as a treatment option for PPD.^32-34^ In 2019, the intravenous administration of brexanolone, a proprietary formulation of allopregnanolone, became the first US-approved treatment for PPD.^34^ However, brexanolone has some limitations, such as the need for hospitalization and the risk of excessive sedation and loss of consciousness; hence, the demand for oral treatment options remains unmet.^34^ Sage Therapeutics and Biogen have developed oral zuranolone (ZURZUVAE™), a novel neuroactive steroid that targets synaptic and extrasynaptic GABA-A receptors as a positive allosteric modulator (PAM) for the treatment of mood disorders. On August 4, 2023, zuranolone was given the nod for treating PPD in adults in the US.^35^ This sanction marks the initial green light for an oral care option to deal with this disorder in the US.^34^

### Pharmacodynamics

Zuranolone is a new synthetic neuroactive steroid known for its selective positive allosteric modulation of the GABA-A receptor.^36^ The mechanism behind the therapeutic benefits of zuranolone for patients with PPD is not completely understood, but it is believed to be related to PAM of GABA-A receptors. In vitro research has shown that zuranolone increases a range of activities in both synaptic and extrasynaptic configurations of human recombinant GABA-A receptor subtypes.^36^ Studies conducted on rodent brain slices have revealed that zuranolone enhances both the tonic and phasic conductance of the GABA-A receptors, which is in line with the heightened surface expression of the receptors. Additionally, zuranolone manifested a considerable PAM of the GABA-A receptors in several in vivo models.^36^

### Pharmacokinetics

The maximum plasma concentration (Cmax) and the plasma concentration-time curve area (AUC) of zuranolone increase proportionally to dosage when taken in the 30-60 mg range. After oral administration, it takes only 5-6 hours for zuranolone to reach Cmax.^37^ When administered once daily, zuranolone accumulates in the body at approximately 1.5 times the administered amount and reaches a steady state after 3-5 days. Administration of 30 mg of zuranolone with a fatty meal increases Cmax and AUC. It should be noted that zuranolone has a high binding affinity to plasma proteins, with a rate of 99.5%. Additionally, its volume of distribution after oral administration exceeds 500 mL.^37^

Zuranolone is primarily metabolized in the liver via the CYP3A4 enzyme. Circulating metabolites are unlikely to have therapeutic effects. Zuranolone has a terminal half-life ranging from approximately 19.7 to 24.6 hours and a mean apparent clearance of 33 L/h. After oral administration, 45% of radiolabeled zuranolone is recovered as metabolites in the urine, while 41% is eliminated in the feces. Less than 2% of total fecal clearance is accounted for by unchanged drug. Zuranolone should not be given to patients with severe hepatic impairment or moderate-severe renal impairment.^37^ In this instance, zuranolone should be taken at a recommended dosage of 30 mg once daily in the evening for a period of 14 days. If used alongside a strong CYP3A4 inhibitor, it could raise the risk of toxicity. When combined with a CYP3A4 inducer, it may reduce the effectiveness of the medication. As such, if taking zuranolone concurrently with CYP3A4 inhibitors, the dosage must be adjusted, while usage of CYP3A4 inducers together with zuranolone should be avoided.^37^

## Clinical Studies

### Postpartum Depression

The efficacy of zuranolone towards PPD was assessed via the SKYLARK^30^ and ROBIN^38^ trials, which were phase 3 studies that took place within the NEST clinical development program. Both trials enrolled women aged between 18 and 45 years who were diagnosed with PPD and had a Hamilton Depression Rating Scale (HAM-D) total score of ≥26 at baseline and experienced a major depressive episode which included the period from the third trimester to the 4th week postpartum. The primary outcome in both studies was the alteration in HAM-D total score on day 15 from baseline. On day 15, a Clinical Global Impressions Improvement (CGI-I) response was assessed by patients who rated their condition as “much improved” or “improved.” These studies were meticulously designed clinical trials that assessed the efficacy of zuranolone in treating PPD.^30,38^

In the SKYLARK study, women who experienced symptoms of PPD were treated with a 14-day course of zuranolone at a dosage of 50 mg once daily, resulting in significant improvement.^30^ For the first 15 days, the zuranolone-treated group (n = 98) showed a significantly higher least squares mean change from baseline in HAM-D total scores compared to the placebo group (n = 97), with this difference being statistically significant (*P* = .001). The study found that there was a difference of –4.0, with a 95% confidence interval between –6.3 and –1.7.^30^ Furthermore, patients treated with zuranolone showed significant improvement in insomnia symptoms from the 3rd until the 45th day of treatment as indicated by their HAM-D insomnia item scores.^39^ Furthermore, self-reported results indicate that there were numerical enhancements that were consistent with progress in the HAM-D total score in evaluations such as the Edinburgh Postpartum Depression Scale score and the 9-item Patient Health Questionnaire (PHQ-9) score, resulting in the preference of zuranolone over placebo.^30^ Participants in the SKYLARK study were randomly assigned to receive zuranolone 50 mg or a placebo once daily in the evening with a meal containing fat and were monitored for 45 days. These findings support the notion that zuranolone is a viable option for treating PPD.^30^

The ROBIN study found that a 14-day treatment of zuranolone 30 mg once daily resulted in significant improvement for women experiencing symptoms of PPD.^38^ Specifically, during the first 15 days, the least square mean (LSM) change from baseline in HAM-D total scores was significantly greater for the group treated with zuranolone (n = 76) compared to the placebo group (n = 74), and this difference was statistically significant (*P* = .003). The study found a statistically significant difference of –4.2 with a 95% confidence interval between –6.9 and –1.5.^38^ Furthermore, there were positive improvements seen in HAM-D total scores from the baseline even on day 3 of treatment initiation (*P* = .03) and these improvements continued until day 45 (*P* = .003). Numerical enhancements in the functional health of mothers who were administered zuranolone in comparison to the placebo group were identified through the Barkin Maternal Functioning Index scores reported by patients at all follow-up time intervals. At day 45, the difference among the groups was nominally significant (*P* = .02).^38^

Post hoc analyses indicate that patients who received zuranolone experienced positive effects on insomnia symptoms and their overall functional health and well-being, as reported by the patients themselves.^40,41^ Patients who participated in the ROBIN study were randomly assigned to receive either zuranolone 30 mg or placebo, both taken once daily with an evening meal for a duration of 14 days. Patients were subsequently monitored for 45 days.^38^ The results suggest that zuranolone represents a viable treatment option for PPD.

### Major Depressive Disorder

The efficacy of zuranolone in treating Major Depressive Disorder (MDD) was assessed through one phase 2 study, MDD-201B, and three phase 3 studies, namely WATERFALL, MOUNTAIN, CORAL, and SHORELINE, within the LANDSCAPE clinical development initiative.

In the WATERFALL examination, a 14-day regimen of zuranolone 50 mg once daily resulted in an improvement of depression symptoms in MDD patients.^42^ On day 15, significant improvements were observed in the zuranolone-treated group (n = 248) compared to placebo (n = 251), as evidenced by the least squares mean (LSM) change from baseline in HAM-D scores. The difference was statistically significant (*P* = .01). Notably, nominally significant enhancements in depression, anxiety, and overall functioning symptoms were also reported on day 15 (*P* ≤ .02) with zuranolone treatment in contrast to the placebo group. These observed benefits persisted until day 42.^42^

In the MOUNTAIN study, we observed a least squares mean (LSM) change from baseline in HAM-D score on day 15 after administering zuranolone once daily for 14 days. The treatment course included placebo (n = 157), 20 mg (n = 159), and 30 mg (n = 166) doses. However, as the results indicate, the study’s endpoint was not met. The study findings demonstrate that administering zuranolone at a 30 mg dosage yielded significantly more positive results than a placebo after day 3 (difference of 6.7 vs. 8.3 with a nominal *P* = .016), day 8 (difference of 7.8 vs. 9.9 with a nominal *P* = .008), and day 12 (difference of 6.7 vs. 8.3 with a nominal *P* = .018) in terms of improvement in HAM-D score from baseline.^43^

Furthermore, the CORAL investigation showed that a course of treatment involving a once-daily dose of zuranolone at 50 mg for 14 days was an effective solution for promptly alleviating depression symptoms in MDD patients.^44^ Additionally, the interim efficacy data from the SHORELINE study indicates that zuranolone could potentially offer treatment benefits to patients with MDD through multiple treatment courses.^45^

In the MDD-201B study, individuals diagnosed with MDD were randomly assigned to receive either zuranolone 30 mg once daily for 14 days (n = 45) or a placebo (n = 44) and followed for 42 days.^46^ On day 15, zuranolone exhibited a significantly greater least squares mean (LSM) change from baseline in HAM-D score compared to the placebo (LSM difference –7.0; 95% confidence interval –10.2 to –3.9; *P* < .001). Improvements in the HAM-D score commenced on day two of treatment initiation and persisted throughout all monitored time periods until day 42.^46^ In a phase two study carried out in Japan, a 14-day treatment routine of 20 mg or 30 mg zuranolone once daily demonstrated efficacy in patients with MDD. On the fifteenth day, a considerable improvement in the HAM-D score was observed when compared with that of placebo.^47^ All the studies indicate that zuranolone has the potential to improve depression symptoms in patients with MDD and respond well to treatment. A meta-analysis study demonstrates the antidepressant effects of zuranolone in patients with MDD, rapidly reducing depression severity by day 3. It was noted that zuranolone was generally well tolerated, with the most common adverse events being somnolence and dizziness. However, it is emphasized that further research is required to confirm the long-term therapeutic efficacy of zuranolone.^48^

### Adverse Effects

Adverse effects associated with zuranolone during treatment were generally mild or moderate in severity in the NEST and LANDSCAPE clinical development programmes.^37^ Side effects were observed in 53.3-66.3% of patients who received zuranolone and 44.6-53.1% of patients who received a placebo.^37^ The most frequently occurring side effects were somnolence, nausea, urinary tract infection, fatigue, diarrhea, and headache. Discontinuation of zuranolone commonly causes somnolence, sedation, and dizziness in patients with PPD and MDD. It was observed that the drug did not cause loss of consciousness, withdrawal symptoms, significant changes in blood parameters, vital signs, or electrocardiograms.^30,38,42-44^ Furthermore, there is no evidence of increased suicidal ideation or behavior associated with the use of zuranolone.^37^ A Phase 1 open-label study specifically assessing zuranolone transfer into breast milk found that the relative infant dose (RID) for 50 mg zuranolone was less than 1%, which is well below the 10% threshold generally considered compatible with breastfeeding.^49^ Despite the low levels of zuranolone in breast milk, the most common treatment-emergent adverse events reported by participants in this study were mild, with dizziness being the most frequently reported.^49^ Given the CNS depressant effects of zuranolone, including somnolence and dizziness, it is important to monitor breastfeeding mothers for these side effects and consider the potential impact on their ability to care for their infants.^49^ The benefits of breastfeeding should be weighed against the mother’s clinical need for zuranolone and any potential adverse effects on the breastfed child.

## Conclusion

Zuranolone stands out as a compound with promising potential as a treatment for PPD. In light of the findings reviewed in this article and important information in the literature, it is thought that zuranolone may have positive effects on PPD. Clinical studies show that zuranolone stands out with its rapid effect potential and short-term use advantages. This is an important feature, especially for conditions that require urgent intervention, such as PPD. A rapid improvement in patients’ symptoms may increase confidence in treatment, which may contribute to increased adherence to treatment. However, more research is needed on the long-term effects and safety of zuranolone. At this point, future clinical trials and long-term follow-up studies may help us understand the full potential of zuranolone in treating PPD. Future directions include the use of zuranolone in combination with other treatment methods, investigating its effects on more specific patient groups, and conducting more large-scale clinical studies to evaluate treatment results. This may allow us to better understand the role of zuranolone in the treatment of PPD and improve treatment options in this area. In conclusion, findings that zuranolone may be a promising alternative in the treatment of PPD should be supported by further research, and how these potential advantages can be used in clinical practice should be examined in more detail.

## Data Availability Statement

The data that support the findings of this study are available on request from the corresponding author.

## Figures and Tables

**Table 1.** General Characteristics of the Studies

Study*	Study Type (Phase, Blinding)	Diagnosis	Sample	Time Endpoint (d)	Dosage (mg/d)	Baseline Severity of HAMD Mean (SD)
Deligiannidis et al^38^	III, DB, RCT	PPD	76/74	45	30	T: 28.4 C: 28.8
Deligiannidis et al^30^	III, DB, RCT	PPD	98/98	45	50	T: 28.6 (2.5) C: 28.8 (2.3)
Clayton et al^42^	III, DB, RCT	MDD	266/ 268	42	50	T: 26.8 (2.6) C: 26.9 (2.7)
Clayton et al^43^	III, DB, RCT	MDD	159/166/157	42	20, 30	T1:25.8 T2: 25.9 C: 25.8
Parikh et al^44^	III, DB, RCT	MDD	212/218	42	50	T: 26.8 (2.51) C: 26.6 (2.58)
Cutler et al^45^	III, openlabel, naturalistic	MDD	687/725	364	30	25.3 (4.1)
Gunduz-Bruce et al^46^	II, DB, RCT	MDD	45/44	42	30	T: 25.2 (2.58) C: 25.7 (2.42)
Kato et al^47^	II, DB, RCT	MDD	82/85/82	99	20, 30	T1:24.8 (2.4) T2: 24.6 (2.2) C: 24.5 (2.1)

C, control group; DB, double-blind; HAMD, Hamilton Rating Scale for Depression; MDD, major depressive disorder; PPD, postpartum depression; RCT, randomized controlled trial; S.D., standard deviation; T, treatment group; T1, the group receiving 20 mg of zuranolone; T2, the group receiving 30 mg of zuranolone. *The general characteristics of the relevant clinical studies in the literature on zuranolone are stated by the authors in the table.
